# Emergence of ferromagnetism due to charge transfer in compressed ilmenite powder using super-high-energy ball milling

**DOI:** 10.1038/s41598-020-62171-z

**Published:** 2020-04-02

**Authors:** Satoshi Ohara, Takashi Naka, Kousuke Sunakawa, Shiro Kubuki, Mamoru Senna, Takeshi Hashishin

**Affiliations:** 1 Joining and Welding Research Institute, Osaka University, 11-1 Mihogaoka, Ibaraki, Osaka, 567-0047 Japan; 2 National Institute for Materials Science, 2-1-1 Sengen, Tsukuba, Ibaraki, 305-0047 Japan; 3 Department of Chemistry, Graduate School of Science and Engineering, Tokyo Metropolitan University, 1-1 Minami-Osawa, Hachi-Oji, Tokyo, 192-0397 Japan; 4 Faculty of Science and Technology, Keio University, 3-14-1 Hiyoshi, Kohoku-ku, Yokohama, Kanagawa 223-8522 Japan; 5 Faculty of Advanced Science and Technology, Kumamoto University, 2-39-1 Kurokami, Chuo-ku, Kumamoto, Kumamoto, 860-8555 Japan

**Keywords:** Materials science, Condensed-matter physics, Magnetic properties and materials

## Abstract

Ilmenite, FeTiO_3_, is a common mineral in nature, existing as an accessory phase in the most basic igneous and metamorphic rocks, for example, it is derived from the upper mantle. Therefore, an understanding of the high-pressure physics of FeTiO_3_ is of fundamental importance in the study of rock magnetization. Here, we provide experimental evidence of lattice compression of FeTiO_3_ powder using super-high-energy ball milling, enabling the very high collision energy of 420 times gravitational acceleration. A sample obtained as an ilmenite- hematite 0.5FeTiO_3_·0.5Fe_2_O_3_ solid solution showed a decrease in molar volume of approximately 1.8%. Consequently, the oxidation state in FeTiO_3_ powder was changed into almost Fe^3+^Ti^3+^, corresponding to 87% Fe^3+^ of the total Fe for FeTiO_3_, resulting in the emergence of ferromagnetism. This new ferromagnetic behaviour is of crucial importance in the study of rock magnetization which is used to interpret historical fluctuations in geomagnetism. In addition, the super-high-energy ball mill can be used to control a range of charge and spin states in transition metal oxides with high pressure.

## Introduction

Ilmenite, FeTiO_3_, is a common mineral in nature, existing as an accessory phase in the most basic igneous and metamorphic rocks. For example, FeTiO_3_ is derived from the upper mantle down to depths of some 400 km and thus pressures of 12–13 GPa^1^. Therefore, an understanding of the high-pressure physics of FeTiO_3_ is of fundamental importance in the study of rock magnetization^2,3^. FeTiO_3_ is a typical transition metal oxide where the partially occupied d shells of the cations allow for a range of charge and spin states. Two types of cation charge ordering exist consistent with O^2−^ anions, namely, Fe^2+^Ti^4+^ and Fe^3+^Ti^3+^, in which Fe has d-electron configurations of d^6^ and d^5^, respectively. The charge transfer excitation between these two oxidation states is known and has been observed in a number of Fe- and Ti-bearing minerals^4^. This results in a rich variation of magnetic, electronics and structural transitions. The Fe^3+^/Fe^2+^ ratios in natural FeTiO_3_ minerals under high pressures, closely representative of the upper mantle pressure condition, have been studied experimentally using a diamond anvil cell in which the rate of Fe^3+^ formation by charge transfer in FeTiO_3_ samples showed a rapid increase from ambient up to 2 GPa and saturated at 40% beyond 2–4 GPa to the highest pressure of 14 GPa^5^.

In a previous study, we reported that the lattice for trigonal FeTiO_3_ powder can be compressed by the high collision energy of 150 gravity using super-high-energy ball milling^6^, whereas little lattice compression was found for FeTiO_3_ using conventional high-energy ball milling^7^. This lattice compression is to be expected for the charge transfer in FeTiO_3_ with high pressure. Therefore, we have investigated the oxidation state of Fe cations in a FeTiO_3_ sample milled at a high-energy collision of 150 G by ^57^Fe Mössbauer spectroscopy (Fig. S1 and Table S1). Although the raw FeTiO_3_ powder only showed the oxidation state of Fe^2+^, i.e., before ball milling the sample, mixed oxygen states of Fe^2+^ and Fe^3+^ were confirmed in the FeTiO_3_ sample after ball milling at 150 G with a Fe^3+^ rate of 15%. In this study, we mill the trigonal FeTiO_3_ powder at a higher-energy collision of 420 G to increase the oxidation state of Fe^3+^ with high pressure. Consequently, the oxidation state in FeTiO_3_ powder was changed into almost Fe^3+^Ti^3+^, resulting in the emergence of ferromagnetism. This magnetic behaviour with high pressure is of crucial importance in the study of rock magnetization which is used to interpret historical fluctuations in geomagnetism^8,9^. Thus, a super-high-energy ball mill can be used to control a range of charge and spin states in transition metal oxides with high pressure, yielding the emergence of a large spectrum of functionalities such as metal-insulator transitions^10^, superconductivity^11^, thermoelectricity^12^, and multiferroicity^13^ as well as magnetism.

## Results and Discussion

**Figure 1:**
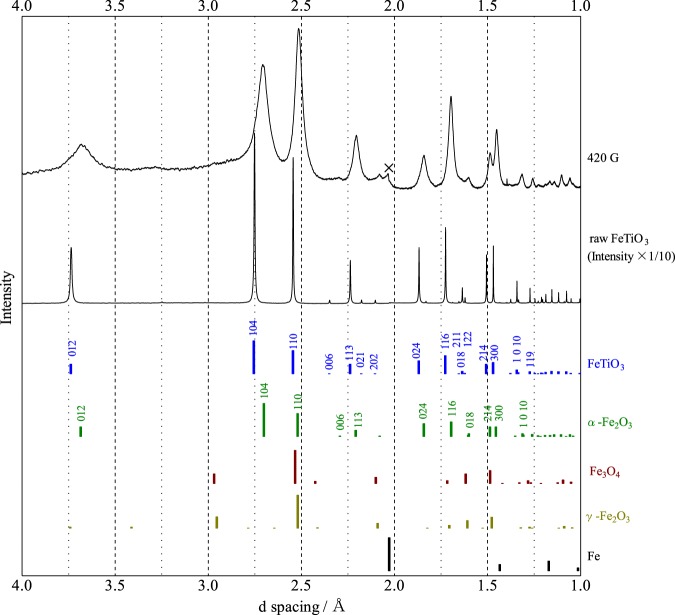
XRD patterns for raw FeTiO_3_ powder and a sample milled at 420 G. The bar graphs are based on the PDF database of XRD patterns; FeTiO_3_: 00-029-0733, α-Fe_2_O_3_: 00-033-0664, Fe_3_O_4_: 00-019-0629, γ-Fe_2_O_3_: 00-039-1346, Fe: 00-006-0696.

The raw trigonal FeTiO_3_ powder, labelled as raw FeTiO_3_, was milled at a very high-energy collision of 420 G (see Methods). The sample was then characterized by measurement of X-ray diffraction (XRD) pattern with synchrotron radiation, as shown in Fig. 1. Notably, the peak position for the as-milled sample was remarkably shifted to a lower d-spacing in comparison with that for the raw powder. This shift in peak position suggests that the trigonal FeTiO_3_ lattice can be compressed by the collision shock between the balls using super-high-energy ball milling, leading to charge transfer from Fe^2+^Ti^4+^ to Fe^3+^Ti^3+^ in FeTiO_3_ with high pressure. Further, the peak position of the as-milled sample was considerably shifted towards a lower d-spacing with increasing collision energy from 150 to 420 G (Fig. S2).

Although metal iron, Fe, was generated from the surface of the steel balls by the super-high-energy ball milling of 150 G^6^ (Fig. S2), no peak due to iron was observed in the FeTiO_3_ sample as-milled at 420 G, as shown in Fig. 1. This absence of iron is considered to be due to the difference in the experimental setup for the ball milling apparatus. The raw FeTiO_3_ powder was milled at 150 G in a closed type steel vial, i.e., under a reduction atmosphere, and at 420 G in an open type steel vial, i.e., under an oxidation atmosphere. Therefore, the formation of haematite, α-Fe_2_O_3_, is presumed to occur via a reaction between oxygen gas in the air and the metal iron generated from the surface of the steel balls by employing an open type steel vial for the ball milling apparatus operated at 420 G. However, it is difficult to identify the formation from α-Fe_2_O_3_ by the XRD pattern (Fig. 1) because both FeTiO_3_ and α-Fe_2_O_3_ crystallize in a corundum-derived structure and they usually exist in natural minerals as solid solutions. On the other hand, other iron oxides such as magnetite, Fe_3_O_4_, and maghemite, γ-Fe_2_O_3_, did not appear as new peaks in the FeTiO_3_ sample as-milled at 420 G. However, one weak impurity peak marked with a cross appeared with an intensity of less than 5% of the main peak intensity, as shown in Fig. 1. The d value for this peak of 2.036 Å did not agree with the first peak for Fe metal (2.027 Å); this remains an unidentified phase.

**Figure 2:**
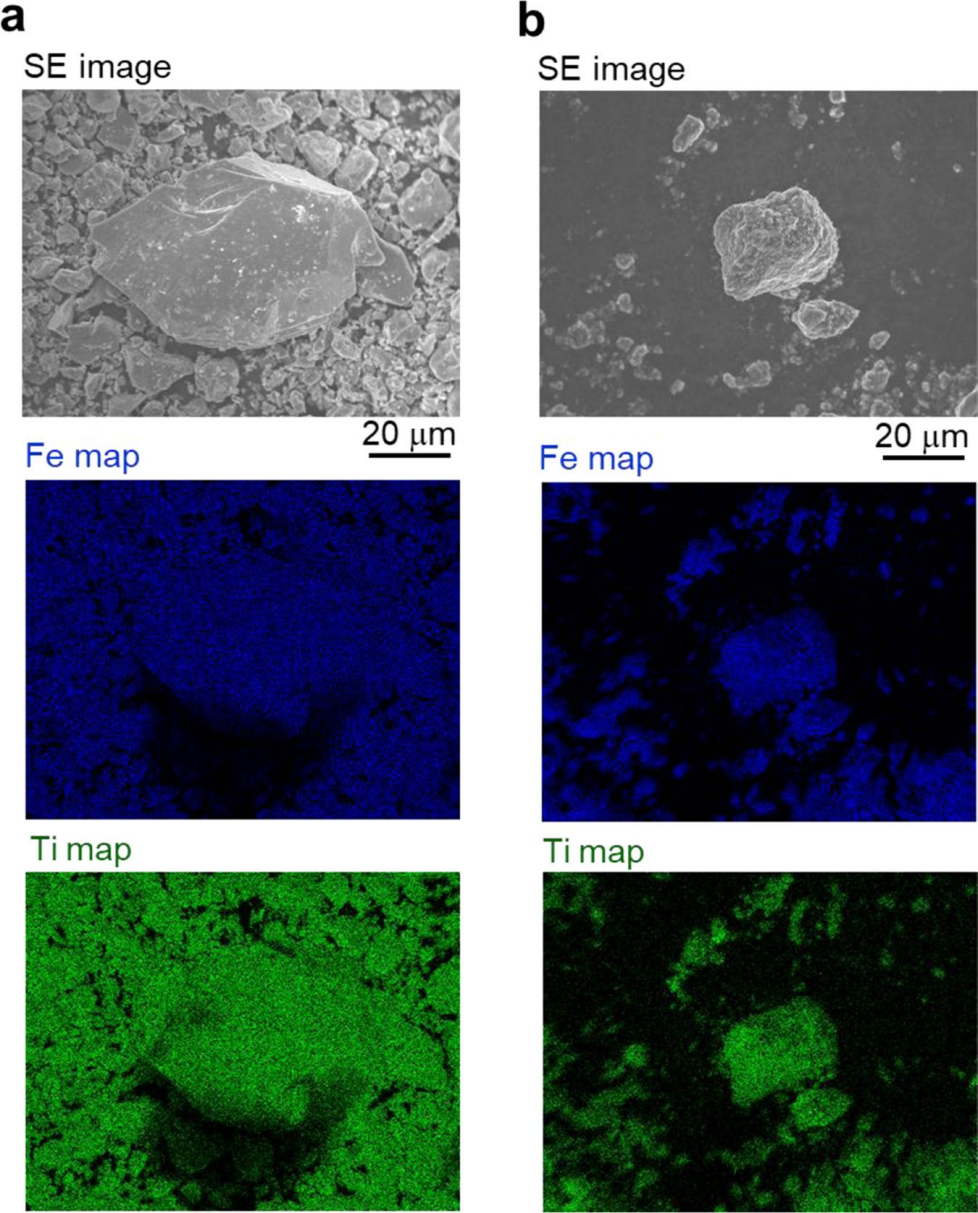
Morphological and compositional feature of raw FeTiO_3_ powder and a sample milled at 420 G. Secondary-electron image and elemental maps for Fe and Ti. (**a**) Raw powder and its compositional ratio of Fe: Ti = 1.0: 1.0 and (**b**) as-milled sample and its compositional ratio of Fe: Ti = 3.2: 1.0.

To clarify the formation of α-Fe_2_O_3_ in the FeTiO_3_ sample as-milled at 420 G, we have investigated the composition of Fe and Ti for the powder sample by an electron dispersion spectroscopy (EDS). The morphological and compositional features of raw FeTiO_3_ powder and an as-milled sample are shown in Fig. 2. Notably, the intensity of Fe in the as-milled sample was stronger that in the raw powder, although the Fe and Ti elemental distribution was very homogeneous for the as-milled sample as well as the raw powder, as shown in Fig. 2. The compositional ratio for Fe: Ti was approximately 3: 1 for the as-milled sample, while the ratio was 1: 1 for the raw powder. Similar results were obtained for another as-milled powder sample by additional EDS analyses (Fig. S3). In general, the FeTiO_3_ shows a solid solution towards α-Fe_2_O_3_ and its chemical composition possesses xFeTiO_3_·(1-x)Fe_2_O_3_. Therefore, it is considered that the ilmenite-hematite (IH) solid solution of 0.5FeTiO_3_·0.5Fe_2_O_3_ was formed and the lattice of the IH solid solution powder was compressed by super-high-energy ball milling at 420 G.

The molar volume for the raw powder and obtained IH solid solution sample determined by least-squares refinement of their XRD patterns was 315.9 Å^3^ and 302.5 Å^3^, respectively, with the value for the raw FeTiO_3_ powder in good agreement with that reported previously^14^. The decrease in molar volume for the IH solid solution powder as-milled at 420 G was approximately 1.8%, which was estimated from the molar volume of 308 Å^3^ for 0.5FeTiO_3_·0.5Fe_2_O_3_ bulk^14^. This percentage of volume decrease due to the very high-energy collision of 420 G corresponds to that of a single crystal of trigonal FeTiO_3_ under a high pressure condition of approximately 5 GPa at 300 K generated using a lever-type diamond anvil cell^15^.

**Figure 3:**
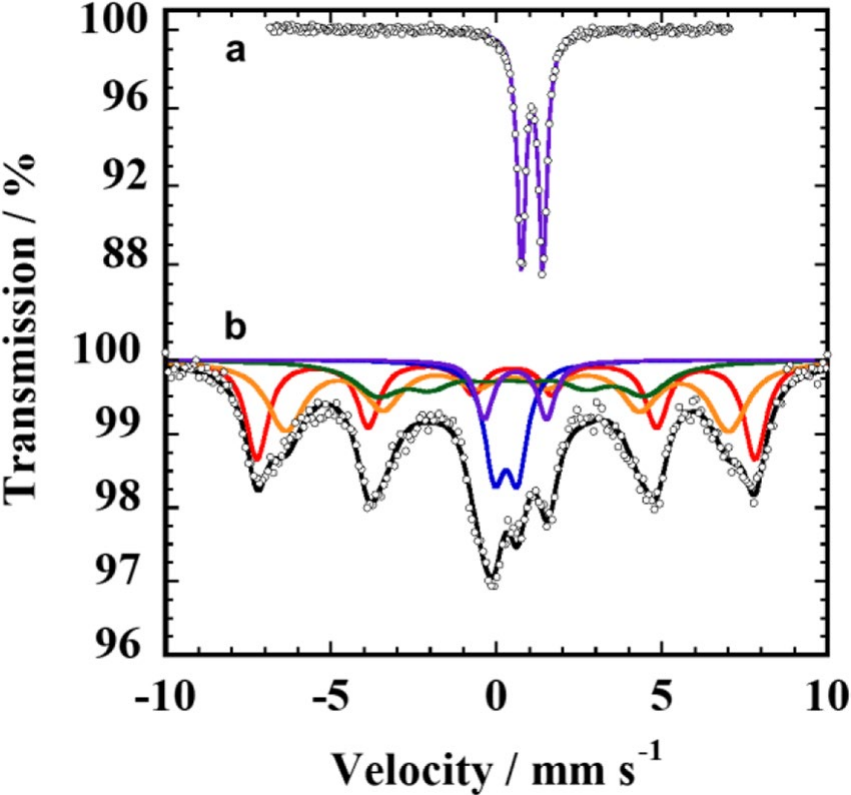
^57^Fe Mössbauer spectra for raw FeTiO_3_ powder and a sample milled at 420 G. (**a**) Raw powder and (**b**) as-milled sample measured at room temperature. The coloured solid lines are fits to the data (see text and Table 1 for details).

Figure 3 shows the ^57^Fe Mössbauer spectra measured at room temperature, with Table 1 listing the corresponding Mössbauer parameters. The spectrum obtained before ball milling showed a quadrupole doublet with an isomer shift, *δ*, of 1.08 mm s^−1^, which is ascribed to Fe^2+^, with the parameters typical for paramagnetic ilmenite^16,17^. On the other hand, the spectrum consisted of two doublets and three sextets fit lines (Fig. 3b) after super-high-energy ball milling at 420 G. The sextet due to Fe metal was not found, which is in good agreement with the result from the XRD pattern. Two sextets, red with *δ* of 0.39 mm s^−1^ and orange with *δ* of 0.40 mm s^−1^ fit lines, are ascribed to Fe^3+^ in the Fe_2_O_3_ component in IH solid solution because their Mössbauer parameters nearly agree with those of α-Fe_2_O_3_^18^. This appearance of two sextets in the Fe_2_O_3_ component seems to be caused by the R$\bar{3}$ space group of the IH solid solution^14^, whereas the α-Fe_2_O_3_ has space group R$\bar{3}$c. These two sextets for the Fe_2_O_3_ component had a total absorption area of 61.2%, which is coherent with the composition of 0.5FeTiO_3_·0.5Fe_2_O_3_ estimated by EDS analysis. Consequently, the residual two doublets and the other sextet are ascribed to the FeTiO_3_ component in the IH solid solution.

**Table 1:** ^57^Fe Mössbauer spectra for raw FeTiO_3_ powder and a sample milled at 420 G.

Species	*A* (%)	*δ* (mm s^−1^)	*Δ* (mm s^−1^)	*Γ* (mm s^−1^)	*H*(T)
**a**					
Fe^2+^	100	1.08	0.65	0.3	—
**b**					
Fe^2+^	4.6	0.78	1.55	0.43	—
Fe^3+^	16.0	0.20	0.92	0.88	—
Fe^3+^	18.2	0.32	0.12	1.63	25.2
Fe^3+^	32.9	0.40	−0.14	1.37	41.6
Fe^3+^	28.3	0.39	−0.21	0.81	46.8

(a) Raw powder and (b) as-milled sample measured at room temperature. SD is less than 0.03 (mm s^−1^) and *A*: ratio (%), *δ*: isomer sift (mm s^−1^), *Δ*: quadrupole splitting (mm s^−1^), *Γ*: line width (mm s^−1^), *H*: Magnetic field (T).

Two doublets, purple with *δ* of 0.78 mm s^−1^ and blue with *δ* of 0.20 mm s^−1^ fit lines, are ascribed to Fe^2+^ and Fe^3+^, with an absorption area of 4.6% and 16.0%, respectively. The sextet, green fit line, with *δ* of 0.32 mm s^−1^ is ascribed to Fe^3+^, with an absorption area of 18.2%. Therefore, the amount of Fe^2+^ for the FeTiO_3_ component in IH solid solution is only 13%, which seems to be a mixed oxidation state with Fe^3+^ because of its *δ* value of 0.78 mm s^−1^. It is found that this large oxidation change from Fe^2+^ to Fe^3+^, corresponding to 87% Fe^3+^ of the total Fe for FeTiO_3_, is caused by the lattice compression of FeTiO_3_ by the super-high-energy ball milling at 420 G. Such very high-energy collision is effective on the oxidation change in Fe cation because of the slight oxidation rate for Fe^3+^ of 5–8%^7^ and 15% (Table S1) for conventional high-energy ball milling and super-high-energy ball milling at 150 G, respectively.

The compression rate for the IH solid solution powder sample obtained by the super-high-energy ball milling at 420 G is approximately 1.8%, which corresponds to that of a single crystal of trigonal FeTiO_3_ under a high pressure condition of approximately 5 GPa generated using a diamond anvil cell^15^. The Fe^2+^ to Fe^3+^ ratio in natural FeTiO_3_ minerals under high pressures has been studied using a diamond anvil cell in which the oxidation rate for Fe^3+^ was approximately 40% at 5 GPa^5^. It is considered that the higher Fe^3+^ rate of 87% obtained by the very high-energy collision of 420 G is achieved by a characteristic of the ball milling process in which the collision is continuously repeated during milling. The unit-cell compression in FeTiO_3_ is quite anisotropic, with the c-axis being more compressible than the a-axis^19^, so that this anisotropic compression seems to take place preferentially for the FeTiO_3_ in IH solid solution powder by repeated very high-energy collisions during the super-high-energy ball milling.

**Figure 4:**
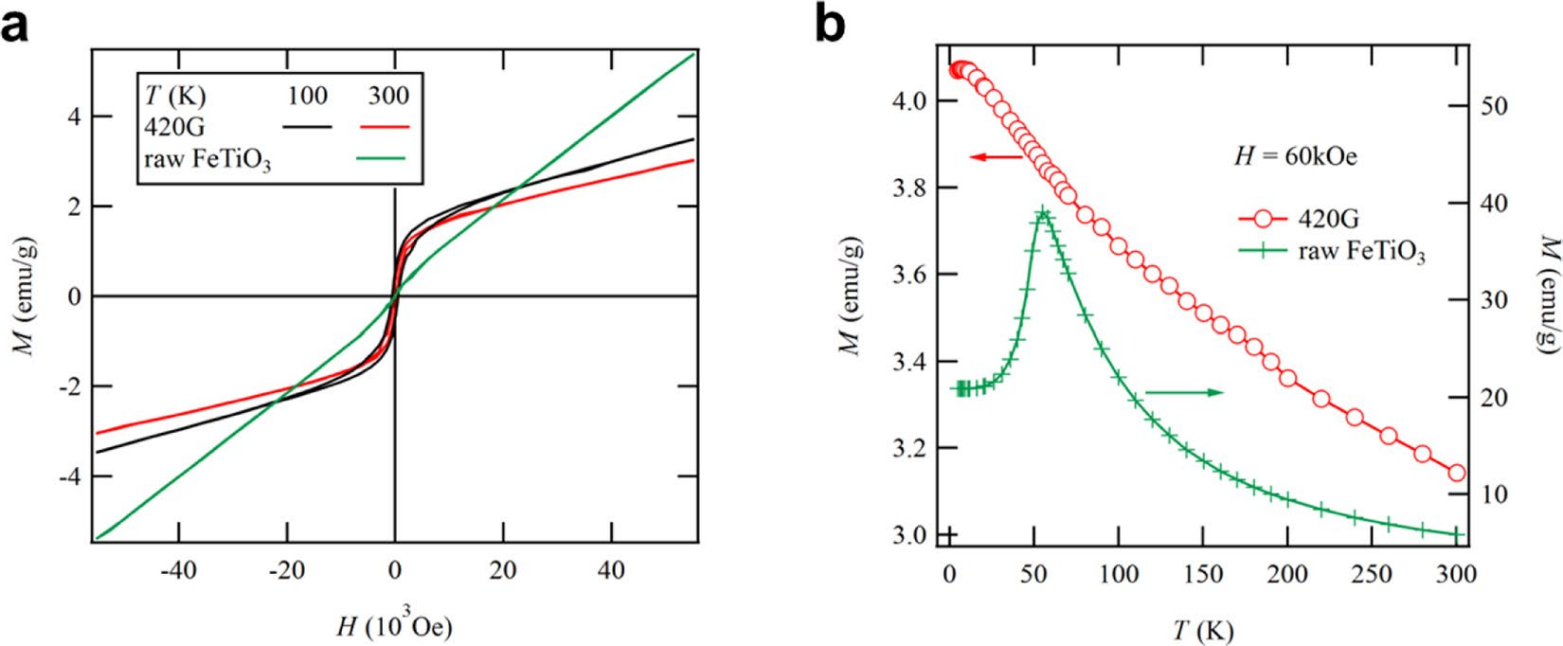
Magnetic properties of raw FeTiO_3_ powder and a sample milled at 420 G. (**a**) Magnetization measured at 300 and 100 K. (**b**) Temperature dependence of the magnetization at 60 kOe.

Notably, the FeTiO_3_ component in compressed IH solid solution possessed the sextet, green fit line, as shown in Fig. 3b. This is to be expected for the emergence of ferromagnetism in FeTiO_3_. Wilson *et al*. have reported computation of the magnetic property of FeTiO_3_ from first principles^20^, and according to them, ferromagnetism is stable in the charge transferred Fe^3+^Ti^3+^ state within the Hartree-Fock approximation. Therefore, we have measured the magnetic properties of the powder sample as-milled at 420 G by a superconducting quantum interference device (SQUID) magnetometer. Figure 4a shows magnetization M as a function of the applied magnetic field H at 300 and 100 K. The as-milled sample showed a mixed magnetic behaviour between ferromagnetism and paramagnetism, whereas the raw trigonal FeTiO_3_ powder showed a paramagnetic state at 300 K. These results are consistent with the Mössbauer spectra (Fig. 3). It is found that the FeTiO_3_ in compressed IH solid solution was weakly ferromagnetic and that its dependence on temperature was negligible. This magnetic behaviour is coherent with the temperature dependence of magnetization, as shown in Fig. 4b.

It is known that IH solid solution exhibiting R$\bar{3}$ symmetry shows large ferrimagnetism in thin films^21–23^ and bulk samples^14,24^. In this case, the cation charge orderings in FeTiO_3_ is Fe^2+^Ti^4+^ without charge transfer and the slope in magnetization versus temperature is quite steep in comparison with that for the powder sample as-milled at 420 G (Fig. 4b). Therefore, it is considered that the ferromagnetism in compressed IH solid solution is not related to the conventional ferrimagnetism in the IH solid solution and that it emerges from the cation charge orderings of Fe^3+^Ti^3+^ by charge transfer in the compressed FeTiO_3_. This ferromagnetic behaviour is of crucial importance to studies in which rock magnetization under high pressure is used to interpret historical fluctuations in the earth’s magnetic field^8,9^. It is now an open question as to the reason why the compressed FeTiO_3_ sample can be quenched by super-high-energy ball milling. It may be due to the charge transfer in FeTiO_3_ because the irreversible nature of charge transfer in natural FeTiO_3_ minerals has been shown in experimental study using a diamond anvil cell^5^.

## Conclusion

In conclusion, we milled trigonal FeTiO_3_ powder using super-high-energy ball milling at 420 G and succeeded in lattice compression of FeTiO_3_ powder. A sample obtained as an IH solid solution showed a decrease in molar volume of approximately 1.8%. Consequently, charge transfer from Fe^2+^Ti^4+^ to Fe^3+^Ti^3+^ took place in FeTiO_3_ with high pressure, resulting in the emergence of ferromagnetism. This finding enables us to infer that such simple and intense collisions induced by super-high-energy ball milling can be used to control the range of charge and spin states in transition metal oxides with high pressure. We believe that property tuning for many functional materials such as graphite^25^ and graphene^26^ with high pressure will be realized and that various high pressure materials^27^ including silica^28^ and titania^29^ with α-PbO_2_ structures will be synthesized based on the super-high-energy ball milling process.

## Methods

### Materials

The raw material was commercially available FeTiO_3_ powder (99.9%, Mitsuwa Chemicals Co., Ltd) with a mean particle size of 149 µm.

### Super-high-energy ball milling

One gram of FeTiO_3_ powder was loaded into a 200 cm^3^ cylindrical steel vial along with 35 g of milling balls. The milling balls were commercial stainless steel balls such as SUS440C, which is a solid solution of iron (Fe, 83 wt.%), chromium (Cr, 16 wt.%), and carbon (C, 1 wt.%) with a diameter of 3 mm. The powder was super-high-energy ball milled using a Thinky Nano Pulverizer (NP-100, Thinky Corporation). The ball milling apparatus operated for 10 min in air atmosphere using an open type steel vial under 420 centrifugal forces, with the ball milling treatment repeated 3 times. It is considered that this short milling time of 30 min is suitable for maintaining the crystallinity of the powder sample. In this experiment, the open type vial was used in order to release the heat in the vial generated by the high collision energy of 420 G.

### XRD measurement

Powder x-ray diffraction patterns were measured for the samples filled inside a capillary with an inner diameter of 200 μm using a synchrotron x-ray source (λ = 0.65296 Å) in the BL15XU beam line at SPring-8, Harima, Japan^30^.

### SEM-EDS measurements

A small piece of the produced powder was suspended in ethanol by ultrasonication until a homogeneous suspension was obtained. The suspension was dropped onto an aluminium sample holder, dried, and examined by scanning electron microscopy (SEM) with electron dispersion spectroscopy (EDS) operated at 15 kV (JSM-7600F, JEOL).

### Mössbauer measurements

We measured the ^57^Fe Mössbauer spectra using the constant acceleration method, using a ^57^Co(Rh) source and an α-Fe a reference. γ-Ray radiation was monitored with a multi-channel analyser (MCA-7700, Seiko EG&G) using 512 channels for each spectrum. The obtained spectra were analysed by Lorentzian fitting using Mösswinn 3.0i XP.

### Magnetic measurements

We measured the magnetic properties using a conventional superconducting quantum interference device (SQUID) magnetometer (MPMS-XL, Quantum Design) under a magnetic field of up to 50 kOe in the temperature range from 5 to 300 K for dc-magnetization.

## Supplementary information

Supplementary Information.
