# Supplementary material for: Emergence of ferromagnetism due to charge transfer in compressed ilmenite powder using super-high-energy ball milling
Source: Sci Rep. 2020 Apr 2;10:5293. doi: 10.1038/s41598-020-62171-z (PMC7118105; doi:10.1038/s41598-020-62171-z)
Supplement: Supplementary file 1 — Supplementary Information. [file 41598_2020_62171_MOESM1_ESM.docx]

Emergence of ferromagnetism due to charge transfer in compressed ilmenite powder using super-high-energy ball milling

Satoshi Ohara^1^, Takashi Naka^2^, Kousuke Sunakawa^3^, Shiro Kubuki^3^, Mamoru Senna^4^, Takeshi Hashishin^5^

^1^Joining and Welding Research Institute, Osaka University, 11-1 Mihogaoka, Ibaraki, Osaka 567-0047, Japan.

^2^National Institute for Materials Science, 2-1-1 Sengen, Tsukuba, Ibaraki 305-0047, Japan.

^3^Department of Chemistry, Graduate School of Science and Engineering, Tokyo Metropolitan University, 1-1 Minami-Osawa, Hachi-Oji, Tokyo 192-0397, Japan.

^4^Faculty of Science and Technology, Keio University, 3-14-1 Hiyoshi, Kohoku-ku, Yokohama, Kanagawa 223-8522, Japan.

^5^Faculty of Advanced Science and Technology, Kumamoto University, 2-39-1 Kurokami, Chuo-ku, Kumamoto, Kumamoto 860-8555, Japan.

Correspondence and requests for materials should be addressed to S. O. (e-mail: (ohara@jwri.osaka-u.ac.jp) and T. H. (e-mail: hashishin@msre.kumamoto-u.ac.jp)

**Supplementary information**

**Figure S1. ^57^Fe Mössbauer spectra for raw FeTiO_3_ powder and a sample milled at 150 G.** (**a**) Raw powder and (**b**) as-milled sample measured at room temperature. The coloured solid lines are fits to the data (see Table S1 for details).

**Table S1. ^57^Fe Mössbauer spectra for raw FeTiO_3_ powder and a sample milled at 150 G.** (**a**) Raw powder and (**b**) as-milled sample measured at room temperature. SD is less than 0.03 (mm s^-1^) and *A*: ratio (%), *δ*: isomer sift (mm s^-1^), *D*: quadrupole splitting (mm s^-1^), *G*: line width (mm s^-1^), *H*: Magnetic field (T). Metal iron, Fe, was generated from both the surface of the steel balls and the inside wall of the steel vial by super-high-energy ball milling at 150 G.

**Figure S2. XRD patterns for raw FeTiO_3_ powder and samples milled at 150 and 420 G.** The bar graphs are based on the PDF database of XRD patterns; FeTiO_3_: 00-029-0733, α-Fe_2_O_3_: 00-033-0664, Fe_3_O_4_: 00-019-0629, γ-Fe_2_O_3_: 00-039-1346, Fe: 00-006-0696.

**Figure S3. Morphological and compositional features of the samples milled at 420 G.** Secondary-electron image and elemental maps for Fe and Ti. (**a**) The compositional ratio of Fe : Ti = 3.1 : 1.0 (left side) and (**b**) the compositional ratio of Fe : Ti = 3.4 : 1.0 (right side).
